# Massive right atrial myxoma presenting as syncope and exertional dyspnea: case report

**DOI:** 10.1186/1476-7120-8-23

**Published:** 2010-06-18

**Authors:** Olga Azevedo, Jorge Almeida, Tânia Nolasco, Rosa Medeiros, Jorge Casanova, Carla Bartosch, João Almeida, Paulo Pinho

**Affiliations:** 1 Cardiology Department, Centro Hospitalar do Alto Ave - Unidade de Guimarães, Guimarães, Portugal; 2 Cardio-Thoracic Surgery Center, Hospital São João, Porto, Portugal; 3 Pathology Department, Hospital São João, Porto, Portugal

## Abstract

Primary heart neoplasms are rare occurring with an estimated incidence of 0.0017-0.19%. Myxoma is the most prevalent primary heart tumor. The right atrium is an unusual localization, occurring only in 15-20% of myxoma cases. We report a rare case of a massive right atrial myxoma causing tricuspid valve obstruction and presenting as syncope and exertional dyspnea. This case illustrates the influence of myxoma's size, position and mobility as well as patient's body posture and respiration to the development of signs and symptoms. Three-dimensional echocardiography proved useful in surgery planning, allowing a better definition of the tumor outline and attachment.

## Background

Primary tumors of the heart are rare, with an estimated incidence ranging 0.0017-0.19%, accounting for < 5% of all cardiac tumors. Approximately three quarters of them are benign and nearly half of the benign tumors are myxomas. About 75% of myxomas arise in the left atrium. Right atrial myxomas are rare as myxomas are estimated to occur in the right atrium in only 15-20% of the cases [1]. The low incidence rate of right atrial myxoma has been reported for decades in several autopsy case series. The Mayo clinic series (1954-1979) included 23673 patients and found 41 patients with primary heart tumors (0.17%). A myxoma was found in 28 of those patients and its location was the left atrium in 17 cases and the right atrium in 4 cases (14%) [2]. More recently, Yu *et al*., in a surgical case series, reviewed 33108 patients submitted to cardiac surgery and found 234 cases with a confirmed diagnosis of primary heart tumor (0.71%). A myxoma was found in 184 patients. However, a right atrial localization was observed in only 12 of those cases (6.5%) [3].

Right atrial myxomas may remain asymptomatic [4] or eventually cause constitutional signs and symptoms, including fever, weight loss, arthralgias, Raynaud's phenomenon, anemia, hypergammaglobulinaemia and elevated erythrocyte sedimentation rate (ESR) [1], due to the production of interleukin-6 [5]. Pulmonary embolism of tumor fragments or thrombus from the tumor surface may also occur, resulting in dyspnea, chest pain, haemoptysis, syncope, pulmonary hypertension, right sided heart failure or death [6]. Less frequently, obstruction of the tricuspid valve occurs, resulting in exertional dyspnea, syncope or sudden death [7,8]. Signs and symptoms depend on size, position and mobility of the tumor, varying with patient's respiration and body posture [1].

Echocardiography became the standard diagnosis technique and surgical removal is the recommended therapy, as it is usually curative [1].

Herein, we present a rare clinical case of a massive right atrial myxoma with an uncommon clinical presentation. The value of 3D-echocardiography to the management of the tumor is discussed.

## Case Presentation

### Clinical Case

**Figure 1:**
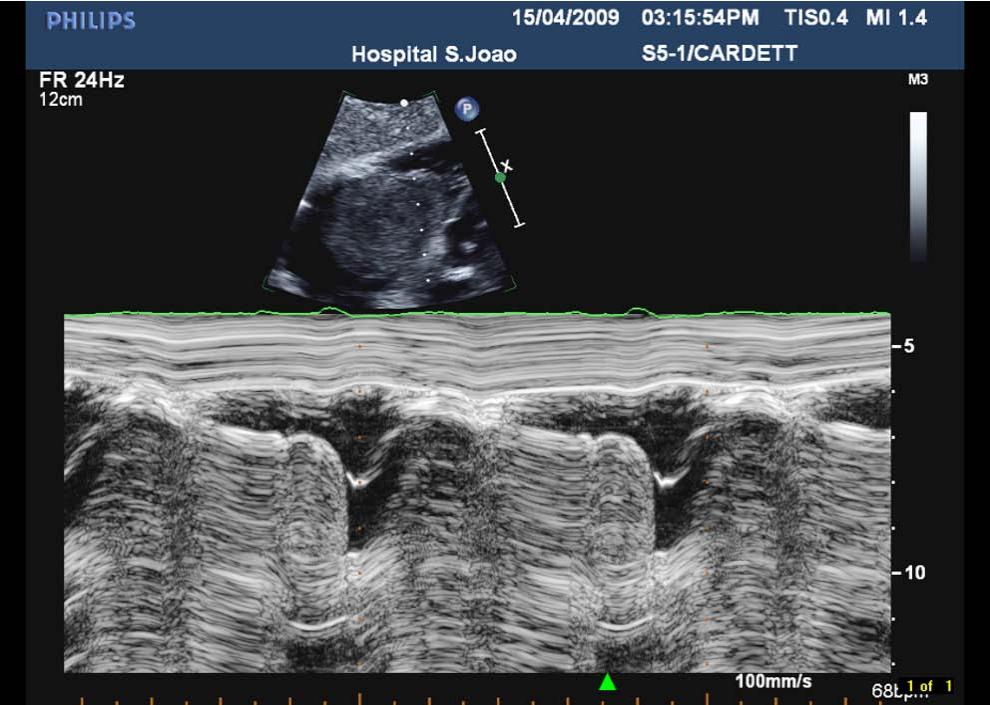
**M-mode of the right atrial mass**. M-mode obtained from a subcostal view showing a huge mass in the right atrium prolapsing into the right ventricle during diastole.

**Figure 2:**
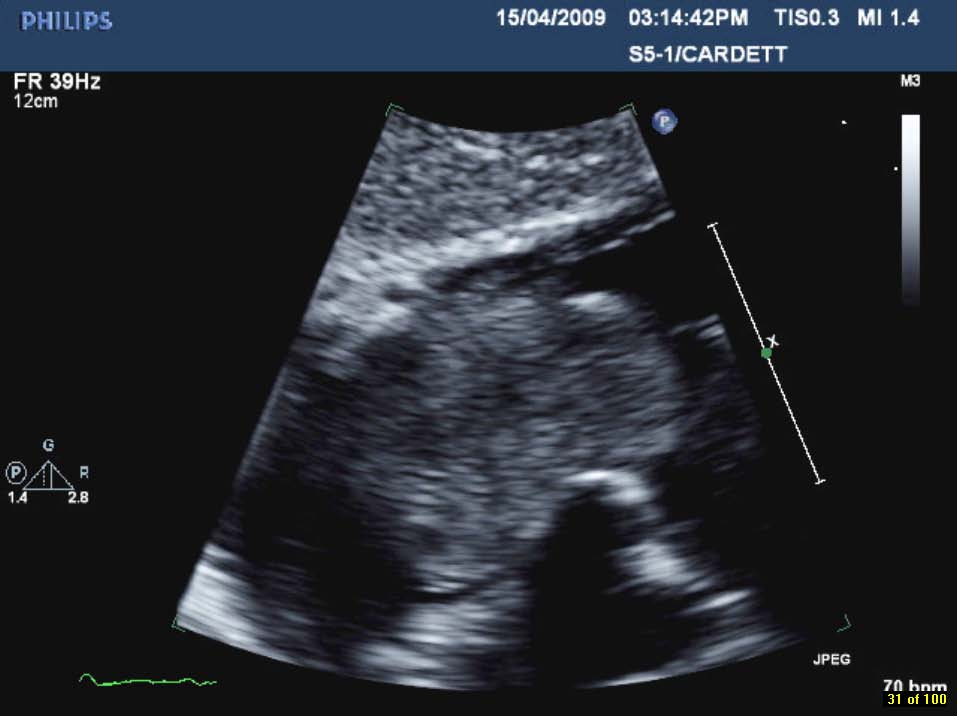
**Right atrial mass on 2D-echocardiography**. A subcostal view showing a huge echogenic mass in the right atrium, prolapsing through the tricuspid valve.

**Figure 3:**
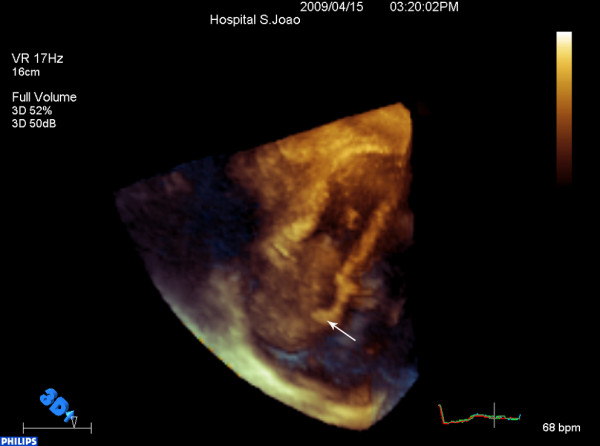
**Right atrial mass on 3D-echocardiography**. 3 D full volume image reconstruction better defined the mass contour and confirmed its attachment (arrow) in the interatrial septum below the *fossa ovalis*.

**Figure 4:**
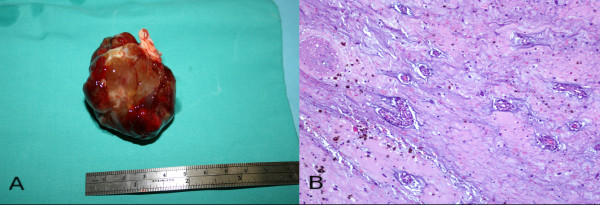
**Histopathological analysis of the atrial mass**. (A) Explanted mass, measuring 4 × 6 cm, and its attachment remnant; (B) Histological section showing the typical myxoid stroma and embedded polygonal cells, blood vessels and areas of hemorrhage (Hematoxylin and Eosin, × 100).

A 74 year-old female, with previous history of hypertension, experienced syncope while performing a vigorous exercise (sawing wood). The development of exertional dyspnea in the next three months motivated her to seek medical attention. Cardiac auscultation was unremarkable. Laboratory data showed an elevated ESR (40 mm). Electrocardiogram was normal. Transthoracic echocardiogram (TTE) revealed an echogenic right atrial mass, of huge dimensions (4 × 6 cm), connected to the lower portion of the interatrial septum by a small pedicle and prolapsing through the tricuspid valve into the right ventricle during diastole (Figure 1 and 2, Additional files 1 and 2). Doppler revealed a mean pressure gradient between right atrium and ventricle of 4 mmHg. Transthoracic 3 D echo allowed a better definition of the mass outline and confirmed the attachment to the interatrial septum below the *fossa ovalis *(Figure 3, Additional file 3). The mass was surgically removed and pathologic analysis confirmed the echocardiographic suspicion of a myxoma (Figure 4). In the follow-up performed two months after surgery, the patient reported a significant symptomatic improvement.

## Discussion

This clinical case is quite unusual, considering the massive dimensions of the myxoma, the uncommon localization in the right atrium and the rare clinical presentation as syncope [8].

In this clinical case, a previously asymptomatic massive right atrial myxoma prolapsing into the right ventricle during diastole probably caused temporary complete obstruction of the tricuspid valve and consequently syncope during a strenuous exercise. Lighter exercises could also have caused lesser degrees of valve obstruction and consequently exertional dyspnea, which is in agreement with the symptomatic improvement reported after the tumor removal. Conversely, in the decubitus position, the cardiac auscultation was unremarkable and the echocardiogram did not find a higher pressure gradient between the right cardiac chambers, which reinforces the role of patient's posture and respiration to the development of clinical signs.

Although TTE provided a good visualization of the mass, 3 D echo proved helpful in surgery planning, allowing a better definition of the tumor outline, position and attachment in the interatrial septum.

## Conclusion

In summary, right atrial myxomas are rare and syncope is a possible although uncommon clinical presentation. This case emphasizes the importance of myxoma's size, position and mobility as well as patient's posture and respiration to the development of signs and symptoms. Right heart tumors should be considered in differential diagnosis of unexplained syncope. TTE is the fundamental diagnostic technique but 3 D echo may also prove helpful in surgery planning, allowing a better definition of the tumor outline, spatial relations and attachment.

## Competing interests

The authors declare that they have no competing interests.

## Authors' contributions

OA, Jorge A and RM drafted the manuscript. TN performed the echocardiogram. Jorge A and JC interpreted the echocardiography data. JC performed the cardiac surgery. CB carried out the pathological analysis. João A and PP reviewed the manuscript. All authors read and approved the final manuscript.

## Supplementary Material

Additional file 1 **2D-echocardiography: subcostal view**. Huge right atrial mass attached to the lower portion of the interatrial septum by a small pedicle, moving back and forth through the tricuspid valve. Click here for file

Additional file 2 **2D-echocardiography: apical 4 chambers view**. Color flow Doppler revealed a mild tricuspid regurgitation. The diastolic flow shows laminar flow through the tricuspid valve, which is in agreement with the pressure gradient between the right cardiac chambers. Click here for file

Additional file 3 **3D-echocardiography**. Live 3 D Echo showing the mass attachment below the *fossa ovalis *and its movement to and through the tricuspid valve. Click here for file
